# Top-Down Control of Visual Attention by the Prefrontal Cortex. Functional Specialization and Long-Range Interactions

**DOI:** 10.3389/fnins.2017.00545

**Published:** 2017-09-29

**Authors:** Sofia Paneri, Georgia G. Gregoriou

**Affiliations:** ^1^Faculty of Medicine, University of Crete, Heraklion, Greece; ^2^Institute of Applied and Computational Mathematics, Foundation for Research and Technology Hellas, Heraklion, Greece

**Keywords:** spatial attention, feature attention, prefrontal cortex, visual cortex, oscillatory synchrony, executive control, long-range interactions, review

## Abstract

The ability to select information that is relevant to current behavioral goals is the hallmark of voluntary attention and an essential part of our cognition. Attention tasks are a prime example to study at the neuronal level, how task related information can be selectively processed in the brain while irrelevant information is filtered out. Whereas, numerous studies have focused on elucidating the mechanisms of visual attention at the single neuron and population level in the visual cortices, considerably less work has been devoted to deciphering the distinct contribution of higher-order brain areas, which are known to be critical for the employment of attention. Among these areas, the prefrontal cortex (PFC) has long been considered a source of top-down signals that bias selection in early visual areas in favor of the attended features. Here, we review recent experimental data that support the role of PFC in attention. We examine the existing evidence for functional specialization within PFC and we discuss how long-range interactions between PFC subregions and posterior visual areas may be implemented in the brain and contribute to the attentional modulation of different measures of neural activity in visual cortices.

## Neural correlates of visual attention

The flexible selection of information that is relevant to current behavioral goals is a critical component of cognition in both human and non-human primates and the hallmark of adaptive behavior. In the visual modality, this function is served by attention. Visual attention facilitates processing of a subset of visual inputs, those that are physically more salient and tend to stand out (“bottom-up” or exogenous attention) or those that are more relevant to behavioral goals based on specific rules or motivation factors (“top-down” or endogenous attention), while irrelevant stimuli are filtered out. Attention can also be distinguished into spatial, when directed to particular locations, and feature or object-based when it is directed to specific visual features (e.g., colors, shapes) or whole objects.

At the neuronal level, most of our current knowledge on the neural underpinnings of visual attention comes from invasive electrophysiological studies in non-human primates and studies that have employed targeted manipulations of neural activity. It is now well-established that attention modulates several measures of neural activity in a way that is consistent with a mechanism that improves the signal to noise ratio (for reviews see Noudoost et al., 2010; Sapountzis and Gregoriou, 2018), thereby increasing signal efficacy for attended stimuli and enhancing the representation of attended locations or features at the expense of distractors.

In the last three decades, several electrophysiological studies have highlighted the effects of attention on neuronal responses of visual cortical neurons. First, attention modulates the firing rate of visual neurons that represent the attended stimulus at different stages of visual processing. Such changes have been reported in subcortical regions (McPeek and Keller, 2002; McAlonan et al., 2008), striate cortex (Luck et al., 1997; Roelfsema et al., 1998; Herrero et al., 2008), extrastriate visual areas (Moran and Desimone, 1985; Treue and Maunsell, 1996; Luck et al., 1997; Chelazzi et al., 1998; Treue and Martinez-Trujillo, 1999; Bichot et al., 2005), parietal (Lynch et al., 1977; Colby et al., 1996; Gottlieb et al., 1998; Bisley and Goldberg, 2003), as well as prefrontal cortical areas (Thompson et al., 1997; Everling et al., 2002; Bichot et al., 2015). This attention-driven firing rate modulation is multiplicative and the scaling of neuronal responses depends on the similarity between the neuron's preferred stimulus and the attended feature (Treue and Martinez-Trujillo, 1999; Martinez-Trujillo and Treue, 2004).

Second, spatial attention leads to a decrease in the variability of responses across trials (Mitchell et al., 2007; Thiele et al., 2016; but see McAdams and Maunsell, 1999) and in the correlated variability (spike count correlations) among neurons (Cohen and Maunsell, 2009; Mitchell et al., 2009; Herrero et al., 2013). This reduction in inter-neuronal correlated variability could potentially improve the signal to noise ratio for attended features and therefore increase the amount of information carried by a neuronal population. Indeed, it has been shown that the decrease in inter-neuronal correlations accounts for most of the improvement in signal quality, whereas attention-related increases in firing rate account only for a smaller proportion (Cohen and Maunsell, 2009; Mitchell et al., 2009).

Third, a number of studies have reported frequency specific modulations in oscillatory synchrony with spatial and feature attention at multiple levels of visual processing (for a review see Gregoriou et al., 2015). These frequency specific modulations of local oscillatory synchrony include an enhancement of local oscillatory activity in gamma frequencies (30–60 Hz) among neurons encoding the attended stimulus (Fries et al., 2001, 2008; Bichot et al., 2005; Tallon-Baudry et al., 2005; Taylor et al., 2005; Gregoriou et al., 2009b; Buffalo et al., 2011, but see Chalk et al., 2010), as well as a decrease with spatial attention in the alpha/beta frequency range (Thut et al., 2006; Fries et al., 2008; Siegel et al., 2008; Gregoriou et al., 2009b; Buffalo et al., 2011). Decreases in local low frequency synchronization, have been associated with inhibition of distracting inputs (Kelly et al., 2006; Palva and Palva, 2007; Handel et al., 2011). On the other hand, an enhancement in gamma synchronization could ensure that neurons, which encode the attended feature, fire together so that their outputs are summed more effectively by downstream neurons (Murthy and Fetz, 1994; Salinas and Sejnowski, 2001; Azouz and Gray, 2003; Fries, 2005, 2009). Such a selective enhancement in local gamma synchronization would result in an increase in signal efficacy for attended stimuli provided that the relative phase of oscillatory activity in the receiving area allows synchronous inputs to be summed effectively by the postsynaptic neurons. Experimental findings, which have shown phase shifted long-range gamma coupling between neuronal populations that encode the attended stimulus, support this hypothesis (Gregoriou et al., 2009b; Bosman et al., 2012; Grothe et al., 2012).

Attention can also change the size and position of visual receptive fields (RFs), bursting activity, response latency (for a review see Clark et al., 2015) as well as feature tuning of neurons (Murray and Wojciulik, 2004; David et al., 2008). How these changes and the aforementioned modulations in different measures of neural activity are brought about remains a fundamental question. Areas in the prefrontal (PFC) and parietal cortex (PPC) have long been considered to be the source of response modulation in posterior visual areas (Desimone and Duncan, 1995; Noudoost et al., 2010; Shomstein and Gottlieb, 2016). Here, we will review the evidence that links PFC to the control of attention. We will focus on studies that have employed electrophysiological approaches and targeted manipulations of neural activity in non-human primates to unravel the causal influences and correlations between activity in PFC and visual cortical areas in attention tasks.

## The role of distinct regions within PFC in spatial and feature attention

If PFC serves as a source of attention-related signals, then neuronal responses in PFC should be modulated by spatial and/or feature attention and prefrontal activity should be causally related to attentional behavior. Moreover, activity in PFC should be correlated and causally related to attention-related activity modulations in posterior visual cortices. In this paragraph, we will review the evidence that activity in PFC fulfills the first two criteria while in the next section (section Does PFC Control the Modulation of Activity in the Visual Cortex?) we will discuss recent findings in support of the last two requirements.

**Figure 1:**
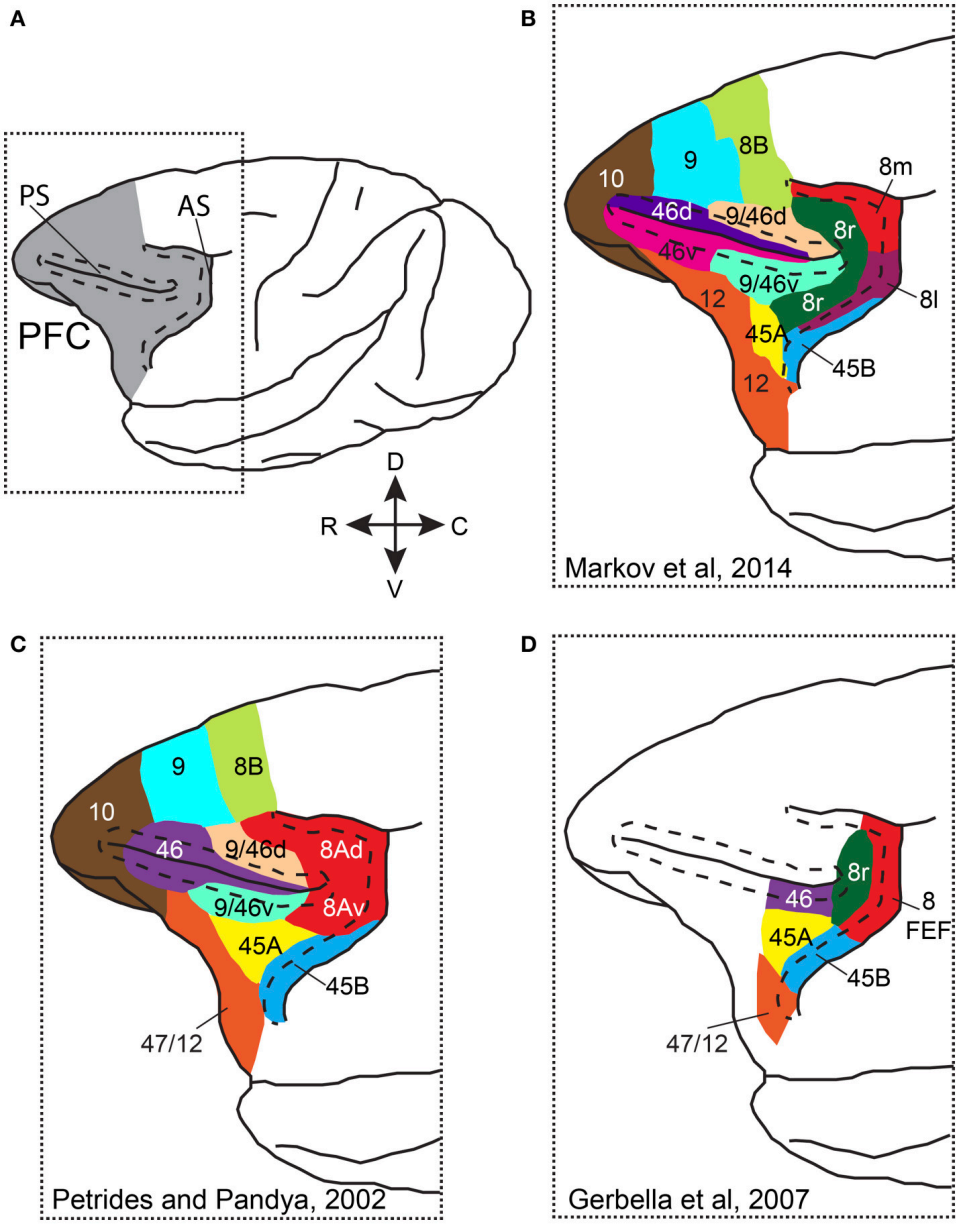
Parcellation and nomenclature of PFC areas in different anatomical studies. **(A)** Dorsolateral view of the macaque brain with PFC colored in gray. Dotted rectangle outlines the area that is shown in **(B–D)**. Dashed line in front of AS follows the lip of the anterior bank of the sulcus, whereas dashed lines around PS outline the dorsal and ventral banks of the sulcus. **(B)** Parcellation of prefrontal areas according to Markov et al. (2014). **(C)** Parcellation of prefrontal areas according to Petrides and Pandya (2002). **(D)** Parcellation of prefrontal areas according to Gerbella et al. (2007). Note that for the different parcellation schemes, the PFC areas are drawn on the same cartoon brain and thus, areal borders and extent are approximate. AS, arcuate sulcus; PS, principal sulcus, C, caudal; R, rostral; D, dorsal; V, ventral.

Anatomically the primate PFC is subdivided into a medial, a lateral and an orbital part, each with its own connectivity pattern (Tanji and Hoshi, 2008). Attention has been more commonly studied in the lateral PFC (LPFC), which comprises areas 8A, 46 (or 9/46), 45A and 45B (Preuss and Goldman-Rakic, 1991; Petrides and Pandya, 1994; Gerbella et al., 2007; Figure 1). Different studies have adopted different nomenclature and parcellation schemes when referring to PFC areas. Figure 1 shows some of the most commonly used PFC parcellation schemes. Whereas, in some anatomical studies the part of the anterior bank of the arcuate sulcus (AS) that hosts the frontal eye fields (FEF) is distinguished from the cortex lying in the convexity anterior to it (Figures 1B,D), in other studies area 8A occupies the anterior bank of AS and extends rostrally on the convexity (Figure 1C). Functional and connectional data also support similar functions for the FEF and the cortical convexity in the immediate rostral vicinity (Moschovakis et al., 2004). Moreover, most electrophysiological studies that have carried out recordings in LPFC, lateral to the principal sulcus, have provided limited evidence on the correspondence of recording sites with anatomically defined areas. As a result, the functional evidence that would support the anatomical parcellation is limited. Nevertheless, most studies distinguish the FEF, which occupies the caudal part of area 8A in the anterior bank of the arcuate sulcus, from the other prefrontal areas and some recent connectional data suggest distinct roles for area 45B, the rostral part of area 8 (8r), area 46, and area 45A (Gerbella et al., 2007, 2010).

The question of whether functional specialization exists within LPFC has been the subject of research efforts for more than 20 years. Early theories suggested a domain specificity in the dorsoventral axis of LPFC, with dorsolateral and ventrolateral PFC processing spatial and non-spatial information, respectively (Goldman-Rakic, 1988; Cavada and Goldman-Rakic, 1989; Wilson et al., 1993; Scalaidhe et al., 1999). Other studies, however, reported that in monkeys trained in cognitive tasks that required the retention of both spatial and non-spatial information, individual LPFC neurons, distributed across different PFC areas, were selective for both types of information (Rao et al., 1997; Rainer et al., 1998; Kadohisa et al., 2013, 2015). More recently, a gradient of stimulus selectivity was found along the rostrocaudal axis of LPFC in passive viewing tasks (Riley et al., 2016). Thus, whether different visual and attentional functions are mediated by distinct PFC subregions or are rather implemented by distinct network signatures that involve the same PFC regions, remains an open question. Here, we will briefly review findings that associate PFC with different forms of attention, discussing the existing evidence on segregation of attention-related functions within PFC.

### PFC and spatial attention

Numerous studies over the last decades, have established a role of PFC in target selection and shifts of attention in spatial attention tasks. Because shifts of attention and shifts of gaze are closely linked in everyday life, structures that are intimately involved in oculomotor functions have been suggested to also control spatial attention.

One cortical area that has mostly been associated with the oculomotor network is the FEF. Early on it was shown that electrical microstimulation of FEF sites with low currents (<50 μA) can evoke saccades of specific metrics (Robinson and Fuchs, 1969; Bruce et al., 1985). Moreover, single-unit studies demonstrated that FEF neurons display visual and/or motor properties during visually and memory guided saccades (Bruce and Goldberg, 1985; Schall, 2002). Later studies established a role for FEF beyond that in saccade production. FEF neurons have visual responses that can distinguish a target from a distractor and thus, reflect the locus of attention both in pop-out and conjunction visual search tasks (Schall and Hanes, 1993; Thompson et al., 1996; Bichot and Schall, 1999; Buschman and Miller, 2007). Importantly, this enhancement in firing rate when the target stimulus is inside the visual RF of the recorded neurons, is independent of the execution of saccades as shown in covert attention (Thompson et al., 1997, 2005; Armstrong et al., 2009; Gregoriou et al., 2009b) and anti-saccade tasks (Murthy et al., 2001; Sato and Schall, 2003). Further support for a distinct role of FEF in attentional functions comes from the fact that only visual and visuomovement and not movement neurons are modulated by spatial attention in the absence of saccades (Thompson et al., 2005; Gregoriou et al., 2012). Interestingly, increases in FEF activity that are endogenously generated (through operant conditioning) result in spatially specific perceptual benefits similar to the behavioral effects of attention (Schafer and Moore, 2011). This result has directly associated enhancements in FEF activity with attention. Thus, rather than simply selecting saccade targets, the FEF appears to have a more general role in highlighting locations of behavioral relevance both in exogenous and endogenous attention. This has led to the proposal that the FEF (albeit not alone) holds a map of attentional priority or salience (Thompson and Bichot, 2005).

Besides the modulation in firing rate, spatial attention has also been associated with changes in local synchrony in the FEF. Attention to a stimulus inside the visual RF of the recorded FEF neurons increases gamma LFP power as well as the coherence between spikes and the local LFP in the gamma (30–60 Hz) frequency range (Gregoriou et al., 2009b). These changes in local synchrony are similar to those reported earlier for visual areas (see above) and could provide a temporal structure to facilitate the selective long-range interactions that will be discussed below.

Addressing, however, whether changes in FEF activity with spatial attention are causally related to target selection and attention, requires targeted manipulations of neural activity. To this end, investigators have employed electrical microstimulation and reversible inactivation of FEF sites. Moore and Fallah (2001) used currents lower than those needed for the generation of saccades to study whether increased activity at particular FEF sites can affect attentional deployment to specific locations. Indeed, monkeys that were asked to detect a luminance change of a target stimulus among distractors showed increased sensitivity during trials in which microstimulation was applied to sites that represented the target location. This was the first demonstration that FEF activity is sufficient to induce improvements in behavioral performance similar to those observed with attention.

Pharmacological inactivation of FEF sites has established that FEF activity is also necessary for normal attentional function. Wardak et al. (2006) showed that reversible inactivation of FEF sites induced spatially specific deficits not only in visually guided saccades but also in covert visual search in both a pop-out and a conjunction task. In the same vein, Monosov and Thompson (2009) demonstrated that suppression of FEF activity leads to spatially selective impairments in a covert visual search task, particularly prominent when an endogenous shift of attention is required. It is thus well-documented that FEF activity is both sufficient and necessary for correct attentional deployment (although FEF is not necessarily the only area with such causal influences). Whether changes in attentional signatures other than firing rate, such as synchrony or inter-neuronal correlations within FEF are also causally related to attentional performance remains to be examined. Indirect evidence in favor of a role of gamma oscillations in PFC for normal attentional processing has been provided by a recent study, which employed optogenetic manipulation of PFC fast spiking parvalbumin interneurons activity in mice (Kim et al., 2016).

The contribution of other PFC regions, besides FEF, to spatial attention has been examined in considerably fewer studies. The existing evidence suggests that spatial attention modulates responses of lateral PFC neurons, in regions extending rostral to the FEF. Neurons that are selective for the location of the target have been described both dorsal and ventral to the principal sulcus (PS) (Everling et al., 2002; Buschman and Miller, 2007; Kusunoki et al., 2010; Lennert and Martinez-Trujillo, 2011, 2013; Kadohisa et al., 2013; Suzuki and Gottlieb, 2013; Bichot et al., 2015), with one study reporting a preponderance of attentionally tuned cells compared to memory-related neurons, ventral to PS (Lebedev et al., 2004). Although most of these LPFC studies have tested spatial attention in conditions that the target is defined in a top-down manner, it has been shown that similar to FEF, LPFC neuronal responses can also discriminate the target location when this is defined by bottom-up factors (Buschman and Miller, 2007; Katsuki and Constantinidis, 2012). Katsuki and Constantinidis (2012), in particular, showed that spatial attention effects in LPFC arise as early as those in PPC in a bottom-up task.

These spatial attention effects can be due to an increase in activity of neurons representing the target and/or a decrease in activity of neurons representing the distractor location. Notably, two studies have underscored a strong effect of distractor suppression in LPFC responses during target discrimination (Lennert and Martinez-Trujillo, 2011; Suzuki and Gottlieb, 2013). The distractor suppression is stronger and longer lasting in LPFC than in PPC (Suzuki and Gottlieb, 2013) and may thus reveal the unique contribution of LPFC in attentional mechanisms. Importantly, some neurons in LFPC acquire selectivity for the location of the target only when distractors are present, a finding in line with a role of LPFC in distractor suppression (Lennert and Martinez-Trujillo, 2013). Consistent with a role in distractor suppression, prefrontal lesions in both human and non-human primates are known to increase distractibility (Chao and Knight, 1995; Gregoriou et al., 2014). Whether similar suppression effects can be found in FEF responses has not been examined in the same tasks; thus, a direct comparison is not possible. It should be noted, however, that enhanced suppression of distractors has also been reported in the FEF, in a pop-out task, following priming of target features (Bichot and Schall, 2002). The relative onset of the suppression effect in FEF and more rostral PFC regions remains to be addressed in future studies.

Although information about the location of the target is carried in PFC neuronal responses, some PFC neurons (as well as neurons in anterior cingulate) encode the instructed location of attention that may differ from the actual locus of attention. Specifically, Westendorff et al. (2016) showed that within PFC, some neurons encoded the spatial information carried by the cue, in trials in which the animal responded to a distractor. This raises the interesting possibility that at least a subset of PFC neurons carries spatial signals that are not used to guide attention online but may instead be used for future behavioral adjustments. Such a finding stresses the importance of examining responses in error trials and has important implications for hypotheses related to the role of PFC as a source of biasing signals to posterior visual cortices.

Studies examining whether an increase in activity in areas of LPFC, outside the FEF, is sufficient to affect behavioral performance in attention tasks are missing. Although this may be due to the larger RFs and the absence of a clear retinotopic organization in LPFC, which makes targeted manipulations harder, the lack of evidence for such a causal relationship begs for future experiments in this direction. One study reported a bias in target selection with electrical microstimulation of dorsolateral PFC sites but these seemed to include low and higher threshold FEF sites (Opris et al., 2005).

Some investigators, however, have directly tested whether LPFC is necessary for normal attention behavior. Rossi et al. (2007) removed PFC unilaterally (including the FEF, areas 8, 9, 45, and 46) in two macaques and transected the forebrain commissures to exclude inputs from the intact hemisphere. The monkeys were tested in a covert attention task. A color cue indicated the target, which was embedded among differently colored distractors. The animals were asked to discriminate the orientation of the target. The authors found that performance was impaired for stimuli contralateral to the lesion when the color of the cue was switched frequently across trials. Performance was normal both when the cue, and thus the identity of the target, was held constant across trials and in a pop-out task. Hence, the PFC lesion impaired the monkeys' ability to switch top-down control. In another study, reversible inactivation of the posterior part of PS rostral to the FEF in monkeys resulted in increased distractibility, consistent with a role of PFC in the suppression of distractors (Suzuki and Gottlieb, 2013). These findings are complemented by human studies, which have shown that PFC lesions impair attention and increase distractibility (Heilman and Valenstein, 1972; Mesulam, 1981; Chao and Knight, 1995).

In sum, both the FEF and more rostral PFC regions have a central role in the control of spatial attention. Distinct contributions and potential differences, however, can only be tested if simultaneous recordings from different PFC regions are carried out in the same task. Only two studies have recorded simultaneously from FEF and more rostral regions and have provided insights into the relative contributions of these areas in attentional mechanisms. In the first study, Buschman and Miller (2007) found that in a pop-out visual search task, neurons in LPFC reflected the location of attention earlier than those in FEF, whereas in a conjunction search task, neurons in both areas signaled the location of the target at comparable times, with the FEF population leading by a marginal difference of 10 ms. These data suggest an earlier involvement of LPFC in pop out search compared to FEF. Unlike other studies, the authors used a cue to inform the subject about the features of the target in the pop-out task. Whether the relative latency of attentional effects would have been different had the authors employed an uncued pop-out task remains to be examined in future studies. Moreover, given the heterogeneity of functional neuronal classes in FEF, which comprise visual, visuomovement and movement neurons, assessing the relative contribution of distinct neuronal types could help bridge results from different studies and clarify the role of FEF in pop-out tasks (Schall et al., 2007). Although there is evidence that attentional selection in FEF precedes that in posterior visual areas not only in conjunction but also in pop-out search (Purcell et al., 2013), future studies employing simultaneous recordings from distinct PFC and posterior visual areas could directly address whether the relative contribution of PFC subregions depends on task demands.

In a follow-up paper, Buschman and Miller (2009) carried out cross correlation analysis between FEF and LPFC pairs of neurons. The results suggested that FEF leads LPFC activity during covert shifts of attention in the conjunction search task. Based on their findings, the authors suggested that FEF is involved in shifts of attention while searching for the target, whereas LPFC in identifying the target once selected and directing behavior to it. This would also be in agreement with the role of LPFC in maintaining behaviorally relevant information in short-term memory (Fuster and Alexander, 1971; Miller et al., 1996) and with the proposed role of dorsal LPFC in the monitoring and manipulation of retrieved information for behavioral guidance (Petrides, 2000), through interactions with posterior sensory areas (Pasternak et al., 2015).

In a second study, simultaneous recordings were carried out in multiple prefrontal regions including the FEF, a ventral prearcuate area (VPA) in the cortical convexity, possibly corresponding to 45A, and a region within the ventral bank of PS (VPS, part of area 46 or 9/46v) (Bichot et al., 2015). Spatial attention effects emerged marginally earlier in the FEF compared to VPA and VPS, and were overall stronger and more widespread in the FEF population compared to the other prefrontal regions. Altogether, the results support the idea that within PFC, activity in FEF is the first to indicate the location of attention and that FEF is therefore a likely source of signals that control changes in neuronal responses in extrastriate areas as will be discussed later.

### PFC and feature-based attention

We commonly use feature-based attention when we search for an object in a cluttered scene. When the features of the target are known in advance, as is the case when we search for a familiar face in the crowd or for our car in a parking lot, we rely on the known features (e.g., color of clothes/car, brand) to locate the target faster. For example, when searching for something red, all red items in the scene become more likely targets. At the neuronal level, attention to specific features (such as color or shape) has been shown to modulate neuronal responses in early, mid- and high level visual areas including V1, middle temporal area (MT), area V4 and the lateral intraparietal area (LIP) (Haenny et al., 1988; Motter, 1994; Treue and Martinez-Trujillo, 1999; McAdams and Maunsell, 2000; Saenz et al., 2002; Martinez-Trujillo and Treue, 2004; Bichot et al., 2005; Ibos and Freedman, 2016; Hembrook-Short et al., 2017; for a review see Maunsell and Treue, 2006). The modulation of responses depends not only on the similarity of the searched-for feature to the stimulus inside the RF but also on the neuron's feature selectivity. Thus, neurons in MT and V4 increase their firing rate for RF stimuli with the searched-for feature only when this is their preferred feature (Martinez-Trujillo and Treue, 2004; Bichot et al., 2005) as predicted by the feature similarity gain model (Treue and Martinez-Trujillo, 1999). Moreover, V1 neurons that are selective for the task-relevant feature show enhanced responses with attention, whereas non-selective neurons for the task-relevant feature are suppressed (Hembrook-Short et al., 2017). In addition to modulating neuronal firing rate, feature-based attention can enhance spike-LFP gamma coherence in visual cortex (Bichot et al., 2005), as does spatial attention. Moreover, inter-neuronal correlations between similarly tuned neurons that are selective for the attended feature are reduced with feature attention (Cohen and Maunsell, 2011), potentially contributing to an enhanced signal to noise ratio for the attended feature. The selective modulation for neurons that encode the attended feature occurs throughout the visual field and together with the spatially restricted modulation induced by spatial attention could contribute to the construction of a map that highlights the locations of likely targets to which behavior can be guided.

The interaction between spatial and feature attention leads to specific predictions on how responses of neurons with particular tuning properties are affected by attention. Spatial attention signals will increase the response gain of neurons with RFs at the attended location, while features other than location will differentially affect the gain of neurons with RFs across the visual field depending on the attended feature and the neurons' feature selectivity. How these selective modulations of responses based on feature selectivity are brought about remains unresolved.

Contrary to spatial attention and given the global effect of feature-based attention across the visual field, no link to the oculomotor system would be expected in principle. Rather, areas that store a representation of the target or relevant feature, an object- or feature- template, are more likely to act as a source of the observed modulations in visual areas. Although PFC areas are likely candidates to store such templates, the evidence for their role in feature attention mechanisms is scarce. Neuroimaging and MEG studies in humans have reported changes in PFC activity with feature attention (Egner et al., 2008; Baldauf and Desimone, 2014). Moreover, an electrophysiology study in macaques revealed that FEF activity reflects the attended feature and that this feature selection mechanism is spatially invariant and occurs irrespective of spatial attention (Zhou and Desimone, 2011). However, unless visual features become behaviorally relevant or the subject has extensive practice with particular visual attributes, FEF neurons show little selectivity for features in the initial part of their visual response or during passive fixation (Schall et al., 1995; Bichot et al., 1996, 2015; Bichot and Schall, 1999; but see Peng et al., 2008). Thus, it is reasonable to ask how these feature attention effects reach the FEF. Areas rostral to the FEF that are feature selective and share direct anatomical connections with the FEF could compute the similarity between the attended feature and a particular stimulus and act as the source of feature attention signals to the FEF and possibly posterior visual areas. Support for this proposal came from a recent electrophysiology study in macaques. Bichot et al. (2015) recorded simultaneously from different prefrontal regions and inferior temporal cortex (IT), in monkeys that were required to memorize the identity of a particular stimulus and subsequently locate it among distractors in a free viewing search task. Neurons in ventral LPFC, within VPA, rostral to the low threshold FEF, were selective for the target and displayed feature attention effects, which emerged earlier compared to those in the FEF, IT and the cortex ventral to the principal sulcus. Going one step further, the authors showed that inactivation of VPA abolished the feature related modulation in FEF responses and impaired search performance for contralateral targets. Thus, this elegant study is the only study to date, which highlights a prefrontal region—VPA—as a likely source of feature attention signals to FEF and possibly other areas.

Based on these results, the emerging picture suggests that areas within LPFC can maintain information about the attended feature and together with FEF guide shifts of attention. Whether feature-related information is transferred from LPFC to FEF in order to construct a priority map to subsequently guide saccades to behaviorally relevant stimuli remains to be proved. The existing physiological evidence together with the well-established intrinsic prefrontal connections (Barbas and Pandya, 1989; Stanton et al., 1993; Yeterian et al., 2012) makes this a possible scenario. Spatial- and feature-related attention signals from the FEF and LPFC could then modulate responses in visual cortical areas in favor of the attended stimulus. In the next paragraph, we summarize the existing evidence that supports this notion.

## Does PFC control the modulation of activity in the visual cortex?

An area that acts as a source of attention-related signals to extrastriate cortex should influence selectively populations of visual neurons with properties related to the attended stimulus. For spatial attention, this can be achieved through long-range excitatory connections between higher order areas that hold a visuotopic map of behaviorally relevant positions and neurons in posterior visual cortices with RFs at corresponding locations. Accordingly, for feature-based attention, neurons in extrastriate areas with similar tuning and selectivity for the attended feature should receive common input from higher order areas that encode the behaviorally relevant inputs. Is there any evidence that PFC areas can act as a source of attention-related signals to posterior visual areas?

Anatomical and functional data support a role of PFC in modulating activity in extrastriate visual areas. The PFC is interconnected with several visual cortical areas (Yeterian et al., 2012) and is thus well-suited to influence visual representations in sensory areas according to attentional demands. Electrophysiological studies have shown that task- and attention-related signals emerge earlier in PFC compared to visual areas (Gregoriou et al., 2009b; Monosov et al., 2010; Zhou and Desimone, 2011; Lennert and Martinez-Trujillo, 2013; Bichot et al., 2015; Siegel et al., 2015; but see how task specific requirements can affect the relative latency of these effects in Khayat et al., 2009; Pooresmaeili et al., 2014). The earlier effects in prefrontal activity are in line with a potential role of PFC in biasing activity in extrastriate cortices either directly or indirectly. However, examining whether PFC activity is causally related to attention-related activity modulations in visual cortex requires more sophisticated analyses and experimental manipulations.

Causal influences of prefrontal activity on visual processing have been documented in both monkeys and humans in memory and visual discrimination tasks (Fuster et al., 1985; Tomita et al., 1999; Barcelo et al., 2000; Monosov et al., 2011). These challenging but enlightening approaches involve the combination of reversible/permanent deactivations or disconnection of prefrontal and visual cortices, with electrophysiological recordings to assess how the absence of PFC activity may affect processing in posterior visual areas. In attention tasks, only a handful of studies have employed such approaches to study the causal influence of PFC activity on neural signatures of attention in extrastriate cortex. These will be reviewed in paragraph Causal Influences. Causal influences of one area upon another can also be assessed indirectly, in a statistical manner, by examining inter-area functional connectivity and directed interactions in the time or frequency domain (e.g., using Granger causality analysis). In the next paragraph, we briefly review the existing knowledge on PFC-extrastriate areas interactions during attention.

### Interactions between PFC and visual cortex

Several decades of research have shown that cognitive functions, such as attention cannot be attributed to a single structure but rather arise from the coordinated activation of neuronal populations across distant brain areas. Thus, understanding the neural mechanisms of attention at the large-scale level requires an appreciation of the neuronal interactions among different brain areas during attentive behavior. To this end, researchers in the last decade have employed simultaneous extracellular recordings from different nodes of the attention network. On one hand, this approach facilitates comparisons across areas in the same animals and behavioral paradigms, and thus helps determine the relative contribution of distinct brain regions in attention. On the other hand, simultaneous recordings across different brain areas is necessary to study dynamic interactions among neuronal populations. Although an increasing number of studies have used this approach, data obtained from simultaneous recordings in attention tasks are still relatively scarce.

Besides showing earlier effects of attention in PFC compared to extrastriate visual areas (Gregoriou et al., 2009b; Monosov et al., 2010; Zhou and Desimone, 2011; Bichot et al., 2015), some studies have directly addressed how long-range interactions between PFC and visual cortex are affected by attention. The hypothesis is that oscillatory coupling between higher order frontal areas that encode goals and current rules and extrastriate areas that process visual information could facilitate the selection of behaviorally relevant information and enhance the representation of attended stimuli in extrastriate areas. Oscillatory activity reflects the rhythmic excitability fluctuations of a neuronal population and creates windows in time during which inputs are more effective in driving the neurons. These temporally constrained windows are the result of rhythmic inhibition within local networks of excitatory and inhibitory neurons (for a review see Gregoriou et al., 2015). Neuronal groups that are rhythmically active can synchronize their activities through phase-locking. This role of phase locking in effective communication was initially formulated as the communication through coherence hypothesis (Fries, 2005; Bastos et al., 2015). Two rhythmically active neuronal ensembles can effectively communicate if the relative phase of the two oscillators allows spikes from one group to arrive at the other group within the temporal window that is most likely to produce spikes. Thus, oscillatory activity could provide a structure that enables effective communication among neuronal populations that encode behaviorally relevant information across distant brain areas. In other words, coordinated oscillations in different neuronal groups could act as the carriers of inter-areal interactions that facilitate selective routing of information among competing inputs (Benchenane et al., 2011). Accordingly, synchronization by phase locking between a prefrontal area and neuronal groups that encode the attended input in extrastriate cortex could mediate the selection process (Baluch and Itti, 2011).

**Figure 2:**
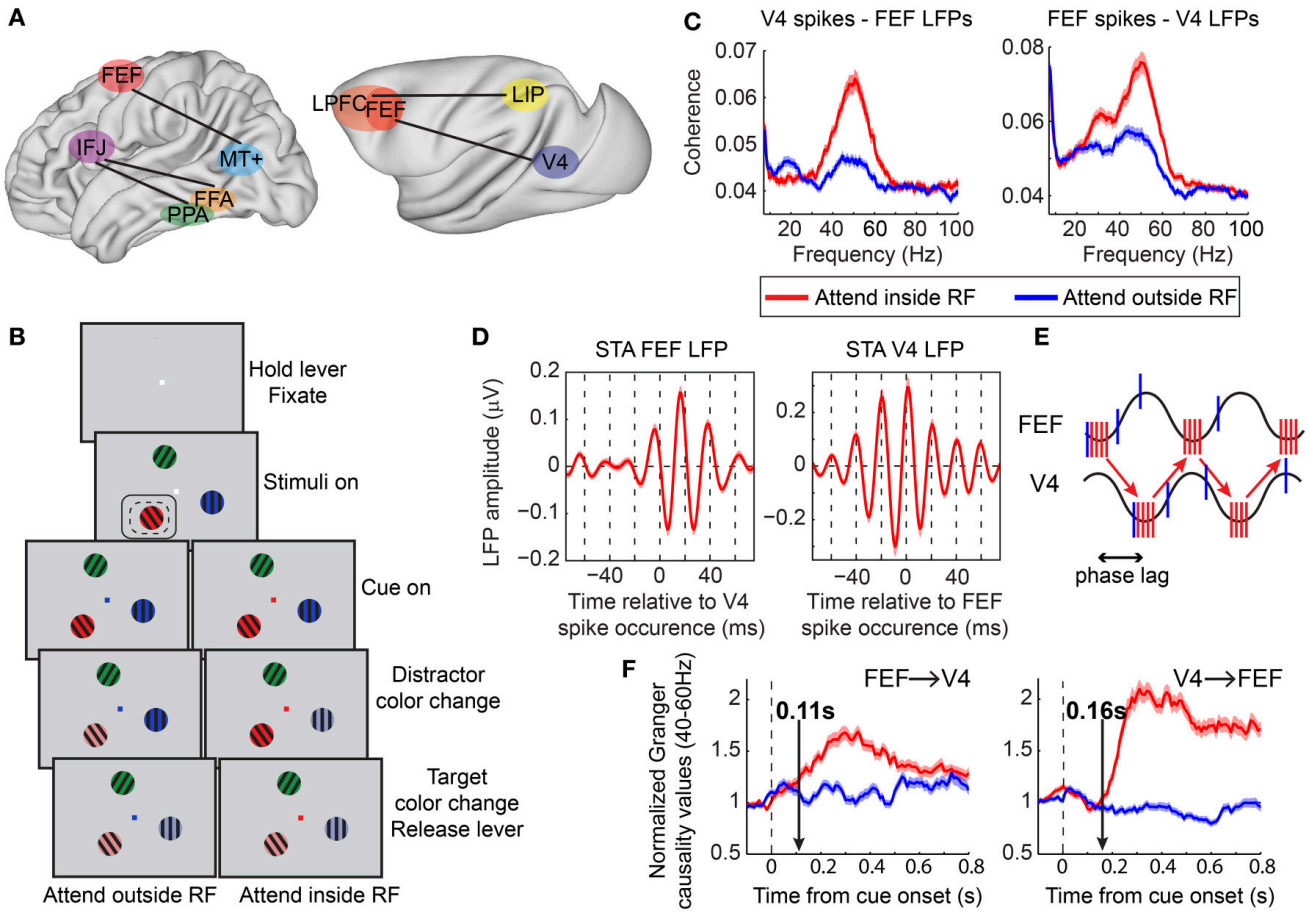
Functional oscillatory coupling between prefrontal and posterior visual areas during attention. **(A)** Schematic of areas involved in long-range oscillatory interactions with attention in the human (left) and in the macaque (right) brain, as described in Buschman and Miller (2007), Siegel et al. (2008), Gregoriou et al. (2009b), and Baldauf and Desimone (2014). Brain surfaces were obtained from the Scalable Brain Atlas website (Bakker et al., 2015). The human brain template is taken from the Harvard-Oxford atlas (original data from Frazier et al., 2005; Desikan et al., 2006; Makris et al., 2006; Goldstein et al., 2007). The macaque brain corresponds to a macaque MRI registered to the F99 space of the Caret software package (Van Essen et al., 2001). **(B)** Behavioral task employed in Gregoriou et al. (2009b). Monkeys had to hold a lever to initiate the trial and were required to fixate a central spot. Subsequently, three sinusoidal drifting gratings of different color appeared on the screen. Monkeys had to maintain fixation of the central spot. The fixation spot was then replaced by a cue whose color indicated the target. The monkeys had to monitor the target covertly and respond by releasing the lever when the target changed color. Potential color changes of distractors had to be ignored. Dashed- and solid-line rectangles represent hypothetical RFs of V4 and FEF sites, respectively. **(C)** Spike-LFP coherence between V4 spikes and FEF LFPs (left) and between FEF spikes and V4 LFPs (right). Enhanced coherence was found in both cases in the gamma frequency range (40–60 Hz) when attention was directed to a stimulus inside the joint V4, FEF, RF (compare red to blue lines). A taper bandwidth of ±7 Hz was used to re-analyze the dataset used in Gregoriou et al. (2009b). **(D)** Spike triggered average (STA) of FEF (left) and V4 (right) LFPs filtered between 35 and 80 Hz. Zero on x-axis corresponds to the time a V4 (left) or FEF (right) spike occurred. Both plots show that spikes in one area tended to occur about half a gamma cycle (about 10 ms) earlier than the time of maximal excitability in the other area (trough in LFP gamma oscillation). **(E)** Schematic description of potential facilitatory effect of inter-areal delays in long-range neuronal communication. Sine waves represent excitability fluctuations in the two areas (gamma oscillations in the LFP). Red and blue vertical lines illustrate action potentials fired for attended (attend inside RF) and unattended (attend outside RF) stimuli, respectively. Spikes in one area that arrive at the phase of maximal excitability in the other area increase the probability of spike generation in the second area (red vertical lines at trough of sine waves). The phase lag between excitability fluctuations in the two areas facilitates this effect for attended stimuli that are associated with coherent spikes fired at the right phase. Less coherent spikes fired for unattended stimuli (blue vertical lines) are not as effective. **(F)** Average of normalized Granger causality values between 40–60 Hz across all V4-FEF LFP pairs. FEF to V4 directional influences are shown on the left graph and V4 to FEF on the right graph. Vertical arrows point to the latency of the attentional effect, which was earlier in the FEF to V4 direction (0.11 s compared to 0.16 s). **(B,D,F)** Modified from Gregoriou et al. (2009b), **(E)** modified from Gregoriou et al. (2009a).

Although limited, supporting evidence suggests that this could indeed be the case. Both beta- and gamma-band synchronization have been implicated in long-range interactions during attention. Buschman and Miller (2007) reported enhanced beta- and gamma-band oscillatory coupling during top-down and bottom-up attention, respectively, between prefrontal (FEF and LPFC) and PPC (LIP) (Figure 2A). Although phase relationships and directional influences between the two regions were not examined, this study suggested that frequency specific oscillatory synchrony might constitute a functional signature of the distinct networks involved in different contexts, a notion that was later extended and has since received considerable experimental support (Siegel et al., 2012; Mejias et al., 2016).

Other studies have shown enhanced gamma band synchronization between prefrontal and visual areas in top-down attention tasks, both in humans and non-human primates. In one MEG study, a spatially specific enhancement in interregional phase coherence was found in the gamma frequency range between the FEF and extrastriate area MT+ in the delay period of a spatial attention task (Figure 2A; Siegel et al., 2008). Similar results were obtained in a covert spatial attention task in non-human primates between FEF and area V4 during sustained attention (Figure 2; Gregoriou et al., 2009b). Enhanced gamma band spike-LFP coherence was found across the two areas between neuronal populations that encoded the attended location (Figure 2C). Importantly, this functional coupling at gamma frequencies occurred at a non-zero phase lag, with spikes in one area occurring ~10 ms before the time of maximal excitability in the other area (Figure 2D). This time lag could correspond to the transmission delays between the two areas and is consistent with a facilitating role of inter-areal delays in long-range communication through coherence (Figure 2E; Bastos et al., 2015). Going one step further, the authors of the study examined directional influences between FEF and V4 using Granger causality analysis. The results suggested that during the early stage of orienting attention to the relevant location, FEF initiates the coupled oscillations across the two areas, whereas the V4 influence on FEF gamma oscillatory activity is more prominent, with overall larger Granger values, during sustained attention (Figure 2F). Given the functional heterogeneity of neuronal types in FEF, it is reasonable to ask whether oscillatory coupling between the two areas pertains to specific cell types. Indeed, Gregoriou et al. (2012) extended their analyses to functionally characterized single neurons in FEF to reveal that only visual, and not visuomovement or movement neurons, show enhanced gamma synchronization with V4 during attention. These data support a role of FEF visual neurons as a source of top-down spatial attention signals that initiate enhanced gamma oscillations in V4 with spatial attention.

Similar coupling between frontal and visual areas has been reported for object-based attention in humans (Figure 2A). In a MEG study, subjects were asked to attend to either faces or houses. Enhanced gamma synchrony was found between the inferior frontal junction (IFJ) and either the fusiform face area (FFA) or the parahippocampal place area (PPA), depending on whether a face or a house was attended, respectively (Baldauf and Desimone, 2014). The pattern of gamma phase lags showed that IFJ led PPA and FFA by 20 ms, suggesting a role of IFJ in initiating gamma oscillatory activity in PPA and FFA during attention for houses or faces, respectively.

Whether inter-areal oscillatory coupling in the gamma band is necessary for PFC to induce an enhancement in firing rate in extrastriate cortex is an open question. Ardid et al. (2010) addressed this question in a modeling study that simulated two coupled networks, a sensory area like MT and an executive control area like PFC, each showing weak oscillatory activity. The authors found that PFC could induce firing rate changes in MT with attention, independent of changes in inter-areal coherence. However, changes in inter-areal synchrony had a major effect on the degree of coherence between neurons that encoded the attended feature across the two areas. These findings suggest that inter-areal gamma synchronization between PFC and extrastriate cortex facilitates selective communication among neuronal populations that encode behaviorally relevant information as previously suggested (Womelsdorf and Fries, 2007), by modestly increasing neuronal responses and enhancing their impact on downstream areas.

Top-down control presupposes the existence of modulatory signals from higher to lower level visual areas that could potentially prime specific representations in visual cortex to facilitate a faster and more effective activation of a particular attentional set. In cases that the subject is cued about the attentional set before the presentation of the stimuli, task related signals should be present during the pre-target period and the top-down influence would be expected to emerge during this preparatory period. This preparatory attention signal from PFC could depolarize sensory neurons in downstream areas so that the latency of stimulus encoding is reduced following stimulus presentation or its salience is enhanced. In that sense, sustained activity in higher order areas in the pre-stimulus period following a relevant cue could be a neural substrate of an attention selection signal (Lara and Wallis, 2015). There is substantial evidence that PFC neurons encode task rules and task selective information in the cue, delay or preparatory period in a variety of tasks (for a review see Sakai, 2008). Moreover, activity in PFC areas has been shown to be correlated and affect, in a task specific manner, activity in downstream regions in the human brain (Sakai and Passingham, 2006; Taylor et al., 2007; Morishima et al., 2009). Electrophysiological studies in macaques have also shown that output neurons in FEF and dorsolateral PFC that are directly connected to the superior colliculus (SC) carry task selective signals in the pre-target period suggesting a way that these areas could influence SC activity (Everling and Munoz, 2000; Johnston and Everling, 2006). Moreover, FEF neurons that project to V4 show enhanced activity during the delay period of a spatial working memory task (Merrikhi et al., 2017). These results support a possible role of sustained activity in driving selection of task specific representations in lower areas. Only few electrophysiology studies, however, have directly examined PFC sustained activity in the pre-stimulus period in attention tasks. Zhou and Thompson (2009) showed that FEF neurons enhance their activity in anticipation of a visual stimulus in their response field in a visual attention discrimination task. Moreover, neurons in VPA encode the attended object during the delay and throughout the search period in a free-gaze visual search task (Bichot et al., 2015). Whether and how sustained attention-related information in these regions is related to activity in earlier visual areas remains to be assessed in future studies.

Preparatory attention could also result in enhanced and faster perception through selective oscillatory coupling between PFC networks and visual cortical areas. Oscillatory synchrony in the pre-stimulus period could again prime neurons selective for the expected feature (location or visual attribute) whereas neuronal populations that code for different attributes would not be primed (Engel et al., 2001). Evidence for such selective coupling during the preparatory period in a spatial attention task comes from an MEG study. Siegel et al. (2008) cued subjects to covertly attend to the right or left visual field. Following a delay period subjects were presented with two dynamic dot patterns, one on each hemifield, and they had to report the perceived motion direction of the cued stimulus. The authors found that interregional synchronization in the gamma frequency range was enhanced between FEF and MT+ during the delay period suggesting selective interactions between the two areas during covert attention shifts in anticipation of stimulus presentation.

### Causal influences

A direct test of whether activity in PFC is necessary and/or sufficient to produce attention-related response modulation in extrastriate areas requires selective manipulations of activity in PFC while monitoring activity in visual cortical areas. The few elegant studies that have employed this approach have deepened our understanding of top-down attentional control by the PFC.

Work by Tirin Moore and colleagues has established that activity in the FEF is sufficient to induce attention-like changes in firing rates in V4. Microstimulation of FEF sites using currents lower than those needed for saccade production enhanced responses of V4 neurons with visual RFs at corresponding locations with the FEF response fields, and suppressed responses of neurons with RFs at other locations (Moore and Armstrong, 2003). Interestingly, the modulation of V4 responses occurred only in the presence of visual stimuli, was stronger in the presence of distractors, and was larger for the preferred stimulus of the V4 recorded neuron. All these effects mimic the influence of spatial attention on the responses of extrastriate neurons, and provide direct evidence that input from the FEF can gate signals in V4 by controlling the gain of V4 visual signals. Similar results were obtained in a study that employed FEF microstimulation and fMRI in monkeys (Ekstrom et al., 2008). The modulation of activity induced in posterior visual areas by FEF stimulation was topographically selective and depended on the presence of visual stimuli and distractors. Moreover, similar to the effect of attention on visual responses, FEF microstimulation increased the contrast sensitivity of neuronal responses in several visual areas (Ekstrom et al., 2009). Thus, both studies solidify the role of FEF as a source of attention related signals to extrastriate cortex and further suggest that bottom-up activation is required for top-down modulation of visual responses by the FEF. It should be noted that this latter requirement contrasts with results in humans and non-human primates, which have shown enhanced activity in visual cortex during top-down attention in the absence of visual stimuli (Luck et al., 1997; Kastner et al., 1999). This discrepancy could indicate differences in the network state induced by microstimulation during a passive fixation task and endogenous attention.

A closer look into the cellular mechanisms of FEF influence on V4 responses was provided by Noudoost and Moore (2011). The authors reported that changes in dopaminergic activity in the FEF can modulate V4 visual responses. Specifically, they injected an antagonist of the dopaminergic D1 receptor within the FEF while recording V4 activity in a passive fixation task. They found that this manipulation induced changes in V4 activity that were comparable to the effects of top-down attention. These included an increase in neuronal responses and orientation selectivity as well as a decrease in trial to trial variability in V4. Moreover, the manipulation of FEF activity led to spatially specific facilitation of target selection in a double target saccade task. Thus, the D1 receptor mediated change in FEF activity was sufficient to induce attention-like effects at both the behavioral and neuronal level, which suggests that modulation of FEF activity through D1 receptors is sufficient to change signatures of attention in V4 through long-range connections. Interestingly, another study by the same group suggested that this long-range influence is exerted mainly by FEF neurons that exhibit persistent activity (Merrikhi et al., 2017), which also tend to show larger attention effects with spatial attention (Armstrong et al., 2009). Other studies have shown that FEF neurons in superficial layers are those that preferentially project to V4 (Pouget et al., 2009) and that activity of visual FEF neurons only is phase locked to V4 activity during spatial attention (Gregoriou et al., 2012). Altogether, these data provide additional insights into the cell type specificity of long-range circuits that underlie attentional mechanisms.

To address whether FEF or other PFC regions are also necessary for the modulation of different attention signatures in extrastriate cortex, three studies have silenced prefrontal areas while monitoring activity in visual cortices. In the first, muscimol, a GABA_A_ agonist, was injected in FEF sites, and activity in V4 was recorded during presentation of visual stimuli in a passive fixation task (Noudoost and Moore, 2011). Inactivation of FEF sites reduced orientation selectivity but had no significant effect on either visual responses or trial-to-trial variability in V4. These results could suggest that FEF is not necessary for activity modulations in V4. However, the absence of competing stimuli in the task employed makes it impossible to draw any conclusions about the role of FEF in the attentional modulation of V4 response magnitude and variability.

To directly examine whether PFC regions are necessary for the attentional modulation of visual processing in extrastriate areas, two other studies employed deactivation of PFC while monitoring activity in posterior visual areas in an attention task. Monosov et al. (2011) demonstrated that following reversible inactivation of FEF, IT responses to the preferred object were significantly reduced when this was presented at locations corresponding to the inactivated sites. Importantly, the effect was more prominent when the neuron's preferred target was presented among distractors. This result suggests that FEF holds an important role in driving object selectivity in IT in cluttered visual displays, consistent with the effect of attention on IT neuronal responses (Zhang et al., 2011).

**Figure 3:**
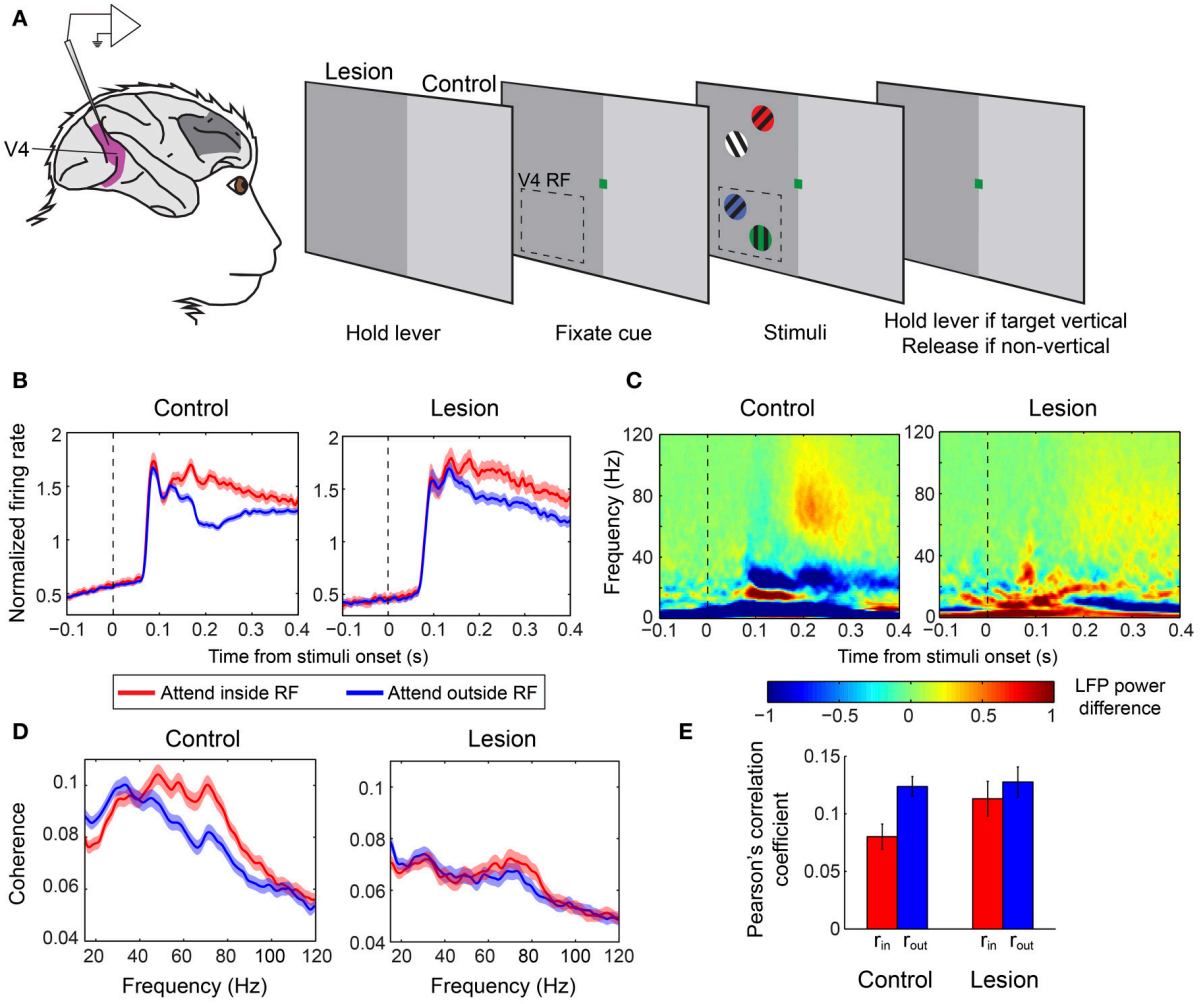
Effect of PFC lesion on the attentional modulation of neural activity in V4. **(A)** Extracellular recordings were carried out in V4 (purple) in monkeys with a unilateral PFC lesion (dark gray patch on monkey brain). Monkeys were required to hold a lever to initiate the trial. Subsequently, a color cue would be presented, which indicated the color of the target stimulus. Four gratings of different colors appeared on the screen, two in the upper (outside the RF) and two in the lower (inside the RF) quadrant. The monkeys were asked to release the lever if the target was a vertical grating and keep holding it if it was non-vertical. Dashed-line rectangle represents a hypothetical V4 RF. When recordings were carried out in the control hemisphere stimuli were presented in the intact hemifield (right half of the screen, light gray), whereas during recording sessions from the lesion-affected hemisphere stimuli were presented in the opposite hemifield (left half of the screen, dark gray). **(B)** Population average V4 firing rates in the two attention conditions from the control (left graph) and lesion affected hemisphere (right graph). Responses are aligned on the presentation of stimuli. Red lines illustrate responses when the target was inside the V4 RF and blue lines correspond to responses when the target appeared outside the RF. Attention effects were significantly smaller in the absence of PFC. **(C)** Time-frequency plots of attentional effects on V4 LFP power (attend inside RF—attend outside RF) in the control (left) and lesion affected hemisphere (right). The attention-induced enhancement in gamma power (60–90 Hz) and reduction in beta power (15–30 Hz) were significantly smaller in the lesion-affected hemisphere. **(D)** Attentional effects on spike-LFP coherence within V4 in the control (left) and lesion-affected hemisphere (right). The enhancement in gamma coherence is significantly smaller in the lesion-affected hemisphere. **(E)** Average Pearson's correlation between spike counts of pairs of V4 neurons (noise correlation) in the control and lesion-affected hemisphere. Red bars correspond to average correlation values with attention inside the RF (r_in_), blue bars correspond to average correlation values with attention outside the RF (r_out_). Error bars indicate mean ± S.E.M. The reduction in noise correlation with spatial attention is significantly larger in the control hemisphere. All graphs **(A–E)** were modified from Gregoriou et al. (2014).

In a second study, Gregoriou et al. (2014) sought to determine whether any region within a larger part of the macaque PFC is necessary for the modulation of neuronal signatures of attention in V4 in a covert attention task. The authors performed a unilateral surgical ablation of PFC including the FEF and regions of the lateral PFC with connections to the ventral visual stream, in two macaque monkeys (Figure 3A). The lesion comprised area 8, dorsolateral areas 9 and 46, and ventrolateral areas 45 and 12, while leaving intact the mesial and orbital prefrontal cortices. To abolish any inter-hemispheric PFC feedback to V4, they also cut the corpus callosum and anterior commissure. As a result, V4 was completely deprived of feedback from PFC in one hemisphere (lesion-affected hemisphere), whereas in the other hemisphere, inputs to V4 from ipsilateral PFC regions were intact (intact hemisphere). The authors recorded neuronal responses in V4 during a covert attention task in the lesion-affected and intact hemispheres, using the latter as a control for the former.

The results highlighted the critical role of PFC in the attentional modulation of different neuronal signatures of attention in V4 including firing rates, neural synchrony and shared inter-neuronal variability. Specifically, in the absence of PFC, the attention-induced enhancement of neuronal responses and gamma synchrony in V4 were significantly reduced in magnitude and were delayed in time (Figures 3B–D). Moreover, LFP power and spike-LFP coherence in the beta frequency range were reduced with spatial attention in V4 in the intact hemisphere, but showed no modulation on average in the lesion-affected hemisphere, indicating that the reduction in beta synchrony within V4 also depends on PFC (Figures 3C,D). The authors also tested whether attention-related changes in shared variability (noise correlation) in V4 depend on PFC. Indeed, the attention-induced decrease in noise correlation reached significance only in the intact hemisphere, pointing to PFC as a major source of feedback signals responsible for the attenuation of inter-neuronal correlations in V4 with spatial attention (Figure 3E). These findings confirm that several different neural signatures of attention critically depend on PFC suggesting that PFC is a source of attentional selection signals to V4 and possibly other extrastriate areas. The authors then hypothesized that if a source of such signals is missing, subjects should be particularly prone to interference by distractors. Consistent with a role of PFC in the suppression of distractors it was shown that in the lesion-affected hemisphere attention was often captured by irrelevant distractors leading to incorrect responses. Importantly, the distractors' effect was reflected on both firing rates and gamma synchrony in V4.

Although the results demonstrate the critical role of PFC in providing top-down attentional modulation of neuronal responses and neural synchrony in V4, they indicate that PFC is not the sole source of such signals in the brain. Given that the attentional effect on firing rates, gamma synchrony and inter-neuronal correlations was not abolished in the absence of PFC, one can conclude that, at least when PFC is missing, other brain regions contribute to the attentional modulation of V4 activity. These could potentially include PPC, prefrontal areas that were spared in Gregoriou et al. (2014), or inputs from the contralateral hemisphere through subcortical structures (for a more detailed discussion see Gregoriou et al., 2014). Whether these brain areas contribute to the attentional modulation of activity in extrastriate cortex also in the normal brain remains an open question. This can only be addressed using reversible deactivation methods, which allow finer temporal control in order to rule out long-term plasticity mechanisms that can lead to functional re-organization of circuits (Jenkins and Merzenich, 1987). Moreover, given the large size of the lesion, which extended across several PFC regions, it is impossible to determine within PFC the exact origin of the top-down influence on V4. Thus, whether the observed attenuation on attentional effects in V4 was due to the absence of FEF, ventrolateral PFC, or periprincipal region needs to be addressed in future studies.

All three studies examined the causal role of PFC regions on extrastriate activity modulations induced by spatial attention. Whether PFC input, however, is necessary and/or sufficient to induce feature-related attentional modulation in visual areas has not been directly examined. Bichot et al. (2015) showed that the reversible deactivation of VPA abolishes feature attention effects in FEF. This result together with data showing earlier feature attention effects in FEF compared to V4 (Zhou and Desimone, 2011) raises the possibility that prefrontal regions anterior to the FEF that process feature specific information, influence extrastriate areas indirectly through FEF in a spatially specific manner. However, a direct test of the causal role of PFC in the spatially invariant feature specific modulations of activity in visual cortex is still missing.

## Conclusions and outstanding questions

The PFC is considered a higher order area that controls several executive functions including attention. The prevailing view holds that PFC neurons encode current goals and rules and facilitate selective processing of information and planning of appropriate actions according to the task at hand. In visual attention tasks, this is implemented by the selective modulation of neuronal responses that represent information relevant to the attended location and feature in posterior visual areas. We reviewed the experimental evidence that supports a role of PFC in gating behaviorally relevant signals in extrastriate areas and we discussed recent findings, which highlight the role of potentially distinct regions within PFC in different aspects of attentional functions. Rather than drawing a complete picture of the circuits and mechanisms that mediate attentional selection, this review aimed to summarize the existing knowledge and highlight the important gaps in our current understanding of how PFC or other brain regions mediate selective visual processing according to behavioral demands. Future studies need to combine anatomical and functional data to directly address whether and how distinct areas within PFC contribute to different aspects of attention. Whether PFC signals are causally related to the spatially invariant, feature-based attention modulation of visual processing in extrastriate areas remains to be examined. Moreover, elucidating the role of different higher order brain areas in attentional processing in the healthy and lesioned brain is expected to extend our understanding of how large-scale networks in the brain contribute to attentional selection. More importantly, optimization of available methodologies should be pursued in order to address the causal role of neural synchrony in different frequency bands in attention mechanisms.

## Author contributions

All authors listed have made a substantial, direct and intellectual contribution to the work, and approved it for publication.

### Conflict of interest statement

The authors declare that the research was conducted in the absence of any commercial or financial relationships that could be construed as a potential conflict of interest.
